# The Logic and Value of the Presumption of *Doli Incapax* (Failing That, an Incapacity Defence)

**DOI:** 10.1093/ojls/gqad010

**Published:** 2023-06-10

**Authors:** David Hamer, Thomas Crofts

**Keywords:** presumption of *doli incapax*, age of criminal responsibility, youth justice, proof beyond reasonable doubt, Crime and Disorder Act 1998, *C v DPP* [1996] AC 1, *RP v R* (2016) 259 CLR 641

## Abstract

Children who do not understand the serious wrongness of their actions lack criminal capacity and cannot be convicted. At common law, children under seven are deemed to lack criminal capacity, children over 14 possess full capacity and children between seven and 14 are rebuttably presumed to lack capacity; the prosecution must prove capacity beyond reasonable doubt. Australia has increased the minimum age of criminal responsibility (MACR) to 10 and is considering a further increase. England & Wales and Northern Ireland have raised the MACR to 10 but have abolished the rebuttable presumption: at age 10, all children are assigned full criminal capacity. This article agrees with international calls for the MACR to be raised but argues that it is more important that the rebuttable presumption should be retained and extended. Children’s brains and decision-making capacities continue to develop throughout their teenage years at different rates. The rebuttable presumption provides individualised justice for children facing developmental difficulties. To wrongfully convict a child who lacks capacity will unjustly damage their life chances. Where a child does have capacity, a variety of evidence may be available to the prosecution to prove it. If the prosecution fails to discharge the burden, the child should be acquitted. The acquittal may be mistaken, but this error is far less harmful than a wrongful conviction.

[I]t is not self-evident that the policy of the law is outmoded in requiring that the prosecution prove the child understood the moral wrongness of the conduct.^1^

## 1. Introduction

For a defendant to be convicted of a criminal offence, they must have criminal capacity. The defendant, at the time of the charged conduct, must have understood that their conduct was seriously wrong.^2^ Adults are taken to have this capacity; however, child defendants^3^ can pose difficult factual issues. Given that capacity is concerned with the child’s mental state, direct evidence will be unavailable. Evidence that appears relatively direct, such as admission evidence or expert psychological evidence, will generally have been obtained some time after the charged conduct and may lack reliability. The child’s capacity develops over time and may be significantly affected by many genetic and environmental factors,^4^ including the charged misconduct and its aftermath. Care will therefore be required in drawing inferences from such evidence.

Where an element of criminal liability poses evidential difficulties like these, the burden and standard of proof play a greater role. The ordinary operation of burdens and standards can be finessed for particular issues through the operation of presumptions. At common law, traditionally, children under seven years of age are irrebuttably presumed to lack criminal capacity.^5^ Between seven and 14 years, incapacity is rebuttably presumed. This presumption, termed the presumption of *doli incapax*, will be rebutted only where the prosecution can bring sufficient evidence to prove ‘beyond reasonable doubt’,^6^ or make the jury ‘sure’,^7^ that the child understood that their behaviour was seriously wrong. As children turn 14 years old, they are irrebuttably presumed to possess criminal capacity.

Over the past few decades, common law jurisdictions have varied the ages over which the various presumptions operate. Many jurisdictions, including England & Wales, Northern Ireland and all Australian jurisdictions, increased the minimum age of criminal capacity (MACR) to 10 years.^8^ At first, England & Wales and Northern Ireland left the rebuttable presumption in place up to age 14; however, this was abolished in 1998. Children are now assigned full criminal capacity at age 10.^9^ Other jurisdictions, such as Canada, Ireland and Scotland, increased the MACR to 12 but also abolished the rebuttable presumption;^10^ at age 12, children are deemed to have full criminal capacity. Regular moves are made in England & Wales to increase the age of criminal responsibility from 10 to 12.^11^ There has also been much debate in Australia about increasing the MACR from 10 years to 12 or 14, with the Territories now beginning to take action.^12^ It appears possible that many Australian jurisdictions will follow the international trend and accompany any increase with the abandonment of the rebuttable presumption.

In this article, we acknowledge the case for the *minimum* age of criminal responsibility to be increased. However, we oppose the abandonment of the rebuttable presumption. Children should not be subject to criminal conviction unless, factually, they committed the conduct with capacity—that is, they did the charged act knowing it to be seriously wrong. Children do not uniformly gain criminal capacity on their 10th birthday, nor on their 14th birthday. This article argues that a more individualised approach is required, differentiating between children according to their level of development. The rebuttable presumption is a logical response to this demand. It is therefore particularly important that the protection of the rebuttable presumption be extended even if the *minimum* age of criminal responsibility is increased. To support this argument, we examine in section 2 the logic of the presumption of *doli incapax* and provide the ‘vigorous [and] reasoned defence’ that, over a quarter of a century ago, Lord Lowry suggested was missing.^13^ Following this, in section 3, we review what sort of evidence can be used to establish a child’s capacity, with the aim of promoting a coherent approach to the use of evidence, and address some of the criticism of the operation of the presumption.

## 2. Raising the Age Is Not Enough

England & Wales, Northern Ireland and all Australian States have a MACR that is considered internationally unacceptable by the UN Committee on the Rights of the Child (UN Committee).^14^ In 2007, the UN Committee in its General Comment No 10 recommended that 12 should be seen as the minimum acceptable level, but that states should work towards a higher age level, such as 14 or 16.^15^ In its General Comment No 24 of 2019, it now encourages states parties ‘to take note of recent scientific findings, and to increase their minimum age accordingly, to at least 14 years of age’.^16^

The UN Committee recommendation is based upon ‘developmental and neuroscience evidence [which] indicates that adolescent brains continue to mature even beyond the teenage years, affecting certain kinds of decision-making’.^17^ The Final Report of the Royal Commission inquiry into the Protection and Detention of Children in the Northern Territory similarly noted: ‘Recent neurobiological research has prompted a reassessment of how recognition of developmental immaturity should affect the way society treats young offenders, particularly in determining the age at which criminal responsibility should be imposed.’^18^ Children ‘are less psychosocially mature than adults in ways that affect their decision-making in antisocial situations’.^19^ They are in a period of neurodevelopmental immaturity, where they are prone to impulsive, sensation-seeking behaviour, with an underdeveloped capacity to gauge the consequences of actions.^20^ Compared to adults, children tend to be less future-orientated with their decisions, give more weight to gains than to losses and have a heightened vulnerability to peer influence.^21^ The UN notes ‘a close relationship between the notion of responsibility for delinquent or criminal behaviour and other social rights and responsibilities (such as marital status, civil majority, etc)’.^22^ In England & Wales and Australia, while 16 or 17 is the age of majority for some purposes (armed services, age of consent, driving), 18 is the age at which people can drink in a pub, smoke and marry.^23^ On this basis, to assign full criminal responsibility to a child at 10, 12 or even 14 appears unacceptable.

While the UN Committee advocates for an increase in the age of criminal responsibility, it does not support the operation of the rebuttable presumption of *doli incapax*. This is because ‘individualized assessment of criminal responsibility … leaves much to the discretion of the court and results in discriminatory practices’.^24^ Rather, states are encouraged to ‘set one appropriate minimum age’,^25^ so that all children below that age are deemed to lack capacity and all above it to possess capacity. But this one-size-fits-all approach is ill-equipped to address the capacity issue. As the Royal Society observes, ‘There is huge individual variability in the timing and patterning of brain development’.^26^ Children of the same chronological age and similar demographic may ‘demonstrate vastly different cognitive capacities for understanding’.^27^ A child’s understanding of the wrongness of misconduct will be influenced by a wide range of interrelated factors, both genetic and environmental, which operate unevenly.^28^ Factors such as child abuse or neglect can ‘impair brain development leading to anxiety, impulsivity, poor affect regulation, hyperactivity, poorer problem-solving and impoverished capacity for empathy’.^29^ Currently, the children most likely to be drawn into the criminal justice system are those experiencing complex needs, such as ‘mental health problems; cognitive disability, including intellectual and developmental disability; physical disability; behavioural difficulties; precarious housing; social isolation; family dysfunction; and problematic drug or alcohol use’.^30^ These factors contribute to the behaviours that lead young people to commit crime: ‘impulsivity, cognitive impairment, alienation and poor emotional regulation’, as well as ‘poor educational attainment, delinquency and illicit drug use’.^31^ In Australia, the rate of developmental vulnerability in Indigenous children is far higher than that of non-Indigenous children.^32^ To impose a single age of criminal capacity, as the UN Committee recommends, would provide formal equality at the expense of substantive equality.^33^ The criminal justice system would be failing the children most in need of protection. The presumption of *doli incapax* is therefore an important tool on top of the MACR to protect children who lack criminal capacity from the criminal justice system.

Below a certain age, say 10 (the current MACR in England & Wales, Northern Ireland and throughout Australia) or 14 (the minimum recommended by the UN Committee), children should be absolutely presumed to lack capacity since virtually all such children do lack capacity. Above a certain age, say 18 (the age at which a person is generally regarded as an adult), people should be absolutely presumed to possess capacity as virtually all such people do possess capacity. In between those ages, where children may either possess or lack capacity, it is appropriate for the prosecution to be required to prove the child defendant’s capacity beyond reasonable doubt, to make the court or jury sure of this for conviction.^34^ This demanding standard of proof minimises the risk of harm to a child from a wrongful finding of capacity. Children that engage in wrongful behaviour without that understanding need care and support at an early stage. It would be a grave injustice to hold them criminally responsible for their actions and deny them that support. Such support should be provided outside the criminal justice system given the clear evidence suggesting that early contact with the criminal justice system is likely to exacerbate their difficulties.^35^

The policy-grounded logic behind the presumption’s burden allocation and adoption of the criminal standard of proof is discussed in section 3. The discussion leads us to advance a reverse-burden defence of incapacity as a backup to the rebuttable presumption. While this would provide children with less protection than the rebuttable presumption of incapacity, it would still enable individualised treatment. In this respect, it is preferable to the abrupt shift from no capacity to full capacity at a specified age. The defence may operate as an alternative to the rebuttable presumption, or as an adjunct for children of older ages, say from 14 to 18 (depending on the age at which the MACR is set).

The UN Committee’s objection to the presumption may be more practical than logical. The Committee’s reference to ‘discretion’ and ‘discriminatory practices’ expresses a concern that court determinations of capacity are inconsistent and unfair. Admittedly, ‘There is no prescribed formula for evidence sufficient to rebut the presumption; that will depend upon the circumstances in individual cases’.^36^ But criminal capacity is hardly unique in that respect.^37^ This problem should not be viewed as insurmountable, precluding individualised capacity determinations. Various types of evidence are commonly available—the child’s age, the nature of the offence, the behaviour of the defendant and the victim at the time of the offence, expert evidence, admissions, and the defendant’s background and upbringing. In section 4, we provide analyses of the inferential and legal issues this evidence presents. If the prosecution is unable to prove capacity beyond reasonable doubt (or, in the case of the incapacity defence, incapacity is proven on the balance of probabilities), the child defendant should be acquitted.

## 3. The Rebuttable Presumption (and/or an Incapacity Defence)

### A. Costs of Error and Standards of Proof

It will be difficult for a court to achieve certainty about a child’s criminal capacity. A major function of evidence law, standards of proof in particular, is managing uncertainty so as to minimise the expected cost of error: ‘the choice of the standard … reflect[s] a very fundamental assessment of the comparative social costs of erroneous factual determinations’.^38^

Consider the demanding criminal standard of proof. There are two possible errors in a criminal trial—convicting the innocent and acquitting the guilty:

[T]he searing injustice and consequential social injury which is involved when the law turns upon itself and convicts an innocent person far outweigh the failure of justice and the consequential social injury involved when the processes of the law proclaim the innocence of a guilty one.^39^

A wrongful murder conviction on the basis of mistaken identity may be viewed as particularly egregious. The defendant had nothing to do with the killing, yet is subject to censure and severe punishment. To minimise the risk of a wrongful conviction, the defendant is presumed innocent and the prosecution carries a heavy burden of proof. This may increase the number of mistaken acquittals, but that price is worth paying to reduce the number of wrongful convictions.^40^

The presumption of *doli incapax* is a relation, or perhaps an instance, of the presumption of innocence. But while the presumption of innocence is, rhetorically at least, the ‘golden thread … always to be seen … and no attempt to whittle it down can be entertained’,^41^ the scope of protection offered by the presumption of *doli incapax* has, in recent decades, been the subject of debate and reform.

Some question whether the presumption of *doli incapax* should operate at all. In *C v DPP*,^42^ Laws J indicated that it is objectionable in principle. ‘It is no part of the general law’ to require proof that a defendant knew that their act was ‘seriously wrong’.^43^ A comparison might be drawn with ignorance of the law, which is commonly said to be no excuse: ‘if a person is alleged to have committed an offence … it is irrelevant to the question of guilt that the accused person was not aware that those elements constituted an offence’.^44^ But such views miss the point. Children have long been recognised as a special case. A child’s ‘ignorance … is natural to every one at that age, and so there is no fault in him … and therefore [he] shall be excused’.^45^ When *C v DPP* reached the House of Lords, Lord Lowry agreed that the presumption was out of step with the general law, but pointed out that ‘the *general* law was not meant to apply without qualification to children under 14’.^46^

Another objection to the presumption of *doli incapax* is that it provides children with special protection that is no longer required; the criminal justice system is not as punitive as in earlier times.^47^ Laws J in *C v DPP* expounded:

the very emphasis placed in modern penal policy upon the desirability of non-custodial disposals designed to be remedial rather than retributive—especially in the case of young offenders, offers powerful support for the view that delinquents under the age of 14 … should not be held immune from the criminal justice system, but sensibly managed within it.^48^

A few years later, in 1998, this attitude informed the British government’s abolition of the presumption of *doli incapax*, assigning children full criminal capacity at age 10.^49^ However, Laws J’s characterisation of modern penal policy as remedial is questionable.^50^ Any suggestion that the criminal justice system provides an appropriate environment for young children seriously underestimates its negative impacts. Indeed, rather than providing rehabilitation, there is the risk that early engagement with the criminal justice system, particularly where it results in detention, ‘can interrupt the normal pattern of “aging out” of criminal behaviour’.^51^ Even without detention, the experience can be traumatising and stigmatising. The difficulties this creates for children completing education or training and gaining employment increases the likelihood they will become chronic adult offenders.^52^ Furthermore, as noted in section 2, the type of child typically drawn into the criminal justice system is likely to face complex needs. If these needs are not addressed early and outside the criminal justice system, they are likely to be exacerbated.^53^

Thus, the need for the presumption of *doli incapax* is to a large extent contingent on the system adopted for responding to children who break the law. If children are to be managed within a criminal justice system that contains the possibility of punitive interventions, they should receive the protection of the presumption of *doli incapax*.

#### (i) Reverse burden defences

Accepting that a finding of criminal capacity should be required for the conviction of a child, the appropriateness of the presumption of *doli incapax* may still be challenged. It could be argued the issue is better handled through a reverse-burden defence, similar to the insanity defence.^54^ Rather than requiring the prosecution to prove capacity beyond reasonable doubt, the defence could be required to prove incapacity on the balance of probabilities. At common law, insanity is the only defence placing this persuasive burden on the defence, but many regulatory offences employ reverse-burden defences.^55^

The lower ‘balance of probabilities’ standard of proof is most commonly used in civil cases. The balanced standard—requiring the claimant to prove their case is ‘more likely than not’^56^—appears appropriate since the claimant and the defendant are generally taken to have comparable stakes in the case.^57^ Unlike in criminal cases, errors either way are generally viewed as equally harmful.

Use of the ‘balance of probabilities’ standard for criminal defences may be justified where erroneously rejecting such a defence appears less of an injustice than other wrongful convictions. Consider the mistaken rejection of insanity in a murder case. Unlike a mistaken identity case, the defence has conceded the killing. Further, the defendant was unlikely to have been freed even had the defence been upheld. The special verdict of ‘acquittal on the grounds of insanity’ would likely have carried severe restriction, supervision and treatment orders.^58^ Erroneous denial of the insanity defence appears less of an injustice than conviction of the wrong person.

A child’s lack of criminal capacity broadly resembles a defendant’s insanity. In both situations, the defence concedes the defendant’s harmful conduct while seeking to avoid criminal responsibility on the basis of mental incapacity; and in both situations, the defendant is likely to face ongoing state supervision even if acquitted.^59^ Despite this, the wrongful conviction of a child who lacks capacity raises graver concerns than the wrongful conviction of an insane defendant. A child’s misconduct from ‘ignorance’ may be considered more ‘excusable’ since it is ‘natural to every one at that age’^60^ rather than the result of mental abnormality. And a convicted child may be more vulnerable to harm from the system. A conviction is likely to seriously damage their opportunity to mature, develop and enjoy a full life. At the same time, ‘fake’ claims of insanity^61^ may raise greater law enforcement concerns than child defendants exaggerating their immaturity and ignorance. As compared with insanity, where a child’s capacity is in issue, a wrongful conviction carries far greater costs than mistaken acquittal. The prosecution should prove a child’s capacity beyond reasonable doubt to minimise the risk of the more harmful error.

Nevertheless, there may be a role for a reverse-burden defence in relation to a child’s capacity. In England & Wales and Northern Ireland, the rebuttable presumption was abolished in 1998, with children assigned full capacity at age 10. In a continuing retributive political environment, it may be more feasible to protect older children by introducing an incapacity defence than by reintroducing the presumption of *doli incapax*. This would give the court some opportunity to recognise that there are child defendants above 10 who lack capacity and address the issue on a case-by-case basis. While not as effective as the presumption, an incapacity defence would still be a big improvement on the current position.

#### (ii) Flexible standards

The reverse-burden defence, incorporating the ‘balance of probabilities’ standard, may appear a blunt instrument with which to take account of relatively less harmful wrongful convictions. Why limit oneself to just two standards of proof, beyond reasonable doubt and balance of probabilities? Flexible standards of proof could provide far greater nuance. While contrary to current authority,^62^ the civil standard could be increased where the claimant makes more serious allegations against the defendant.^63^ And theoretically, ‘there may be degrees of proof within [the criminal] standard’.^64^ Such variation may be difficult to reconcile with the current formulation of the criminal standard in England & Wales, that the fact-finder is ‘sure that the defendant is guilty’.^65^ However, the traditional ‘beyond *reasonable* doubt’ formulation may invite individualisation: ‘a reasonable doubt is a doubt which a particular jury entertain in the circumstances’.^66^ Convicting in the face of a small but tangible doubt may appear wholly inappropriate where the charge is murder, but quite acceptable where the charge is a minor traffic offence.

On this view, the assessment of comparative error costs could vary depending upon the element or defence involved—identity versus insanity—and also with regard to the nature of the offence—murder versus a traffic offence. There could also be variation between cases with regard to the same element of the same offence. Such variation may be more a feature of some elements than others. Identity is all or nothing—the defendant either was the perpetrator or was not. However, criminal capacity lies on a spectrum. The child defendant may have a level of awareness of the wrongness of their actions that falls short of the required level by differing degrees. The injustice constituted by a mistaken finding as to the defendant’s capacity may be in proportion to the shortfall in the defendant’s understanding of the wrongfulness of the conduct. The standard of proof demanded may track this variation. In a case where conflicting bodies of evidence point sharply in two different directions, one where the child defendant had a well-developed appreciation of the wrongness of their actions and the other where the defendant had no idea, the fact-finder may demand that the former evidence is far more credible than the latter in order to convict. In another case, where the evidence is more unified but leaves the fact-finder uncertain as to whether the defendant’s level of understanding quite meets the threshold, the fact-finder may be prepared to convict in the face of a higher level of doubt.

Flexible standards of proof present challenges. To what extent should the circumstances of individual parties be considered as opposed to the abstract legal nature of the claims?^67^ Variability brings with it the risk of inconsistency and injustice.^68^ It is not surprising that recent authorities, particularly in civil cases, have rejected variable standards. Individualised justice may need to be limited in the pursuit of certainty and predictability.^69^

However, even in the face of legal disapproval, the logic of variable standards may be embedded in the psychology of the fact-finder. According to the law of negligence, in determining whether to ‘take precautions against a risk of harm’, a ‘reasonable person’ should take account of the ‘probability’ of harm that may result and its ‘likely seriousness’.^70^ It appears reasonable for a fact-finder to demand a higher degree of persuasion where a wrongful conviction would constitute a graver injustice. For the law to preclude this calculation may leave the fact-finder in a quandary. Of course, the position in England & Wales and Northern Ireland is still more inflexible. There is currently no way for a fact-finder to avoid convicting a child over 10 on the ground of incapacity short of a ‘perverse’ acquittal.^71^

### B. Relative Frequencies and Prior Probabilities

The standard of proof is just one of three factors that contribute to a factual determination. The others are the prior probability of the fact and the weight of the evidence going to the fact. The prior probability—prior to the assessment of any specific evidence relating to the fact—is based primarily on the fact-finder’s view of the inherent (un)likelihood of the fact given their background knowledge and other general circumstances. While reliable statistical data may be unavailable, likelihood is closely related to the assumed relative frequency of such events. For example, the presumption of sanity reflects the ‘common knowledge that a great majority of people are sane, and the probability that any particular person is sane’.^72^

Whether the gap between the prior probability and the standard of proof is bridged depends upon the third consideration—the weight of evidence that is admitted.

[T]he inherent probability or improbability of an event is itself a matter to be taken into account when weighing the probabilities and deciding whether, on balance, the event occurred. The more improbable the event, the stronger must be the evidence that it did occur.^73^

A seemingly demanding standard of proof may be relatively easy to meet if the fact-finder starts with a high prior probability and/or views the evidence as material and credible. Having discussed the standard of proof in the previous section, this section examines the role of the prior probability in rebutting the presumption of *doli incapax*. Section 4 considers the admissibility and weight of various types of evidence.

Prior probabilities may vary depending on the fact in issue. According to leading decisions in Australia and the UK, it may be more demanding to prove serious allegations in civil cases, not because the standard of proof increases, but because the prior probability decreases.^74^ Fraud is more difficult to prove than breach of contract or negligence because of the ‘conventional perception that members of our society do not ordinarily engage in fraudulent or criminal conduct’.^75^ ‘[T]here is no necessary connection’,^76^ but generally speaking, ‘the more serious the allegation the less likely it is that the event occurred’;^77^ greater divergences from normal conduct are less frequent.

At the commencement of a criminal trial, in respect of most elements, the defendant is presumed innocent, and the prosecution bears a heavy burden of proof. As well as the high criminal standard of proof, a low prior probability may also contribute to the weight of the prosecution’s burden. Murder, for example, is a rare and improbable occurrence. Without evidence that the murder occurred and that the defendant did it, the probability of the defendant’s guilt is very low—the presumption of innocence in this respect is factually accurate.^78^

Some commentators argue that the presumption of innocence is a fiction because the prosecution would not have pursued charges without a reasonably strong case; most defendants are convicted, and so any given defendant is probably guilty.^79^ However, while these observations may be correct from a broader perspective, they do not represent the trial court’s perspective. Rather than characterising the trial court’s *prior* probability, these observations make predictions about the evidence that is likely to be presented and the *posterior* probability that may flow from it. It would be illogical to base the *prior* probability on a *prediction* of the prosecution’s incriminating evidence; first, the evidence may not be admitted, and second, if it is, the evidence would then, in effect, be double counted.^80^

The importance of the prior probability should not be overstated. A remarkably low prior probability, based on what is assumed to generally be the case, can quickly be overcome by evidence regarding what occurred in this specific case.^81^ A charged murder would be improbable in the absence of evidence; however, evidence of the victim’s body with multiple gunshot wounds to the head will make it highly probable a murder did occur. Prior to identity evidence, the defendant’s guilt may remain improbable; it could have been anyone. However, this low prior probability may readily be overcome by evidence that the defendant was one of very few people with motive, opportunity and means.

With reference to capacity, the prior probability issue raises psychological questions about the moral development of children and how these relate to legal principle. This article is more concerned with the logic of proof than empirical questions; however, as discussed in section 2, a child’s understanding of the wrongness of misconduct will be influenced by a numerous interrelated factors, and children vary widely in the timing of their development.^82^ The abrupt reversal, in English & Welsh and Northern Irish law, from incapacity to full criminal capacity on a child’s 10th birthday is wholly disconnected from this research. As Smith comments, this ‘holds that a person is completely irresponsible on the day before his tenth birthday, and fully responsible as soon as the jelly and ice-cream have been cleared away the following day’.^83^ Like other irrebuttable presumptions, the assignment of capacity on a child’s 10th birthday has no connection with ‘objective reality’: ‘what is true in the eyes of the law is not directly linked to what may be true in “fact”’.^84^

As Murphy J notes, ‘Conclusive presumptions are dangerous; they prevent any judicial investigation of the matter conclusively presumed, even where it is disputed’.^85^ However, ‘rebuttable presumptions of fact, are useful, and almost indispensable for the operation of an efficient legal system’.^86^ The rebuttable presumption of *doli incapax* is based on a plausible view of reality. Reliable statistical data are unavailable, but it appears reasonable to suppose that, below a certain age, virtually all children lack capacity; these children are irrebuttably presumed to lack capacity. Children do not ‘mature at a uniform rate’;^87^ the proportion or relative frequency of children with capacity can be assumed to gradually increase, reaching almost 100% at a certain higher age. Children above the higher age are irrebuttably presumed to possess capacity. Between these ages, children are rebuttably presumed to lack capacity; however, the prior probability increases with age so that ‘the nearer the child in question is to the [higher] age … the less strong need the evidence be if the presumption is to be rebutted’.^88^

#### (i) Presumption reversal?

**Figure 1.**
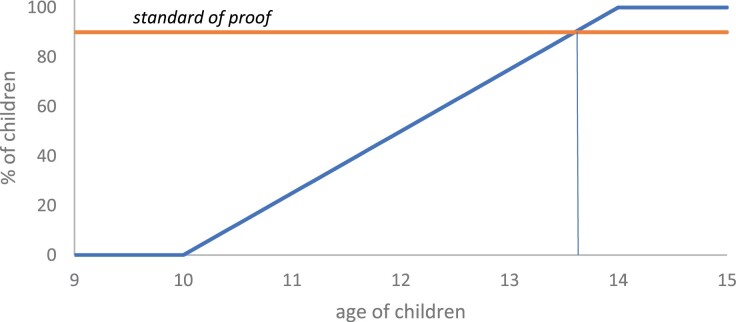
Theoretical construct of percentage of children with capacity: linear distribution.

In relating the presumption of *doli incapax* to notional frequencies and prior probabilities, an interesting wrinkle emerges—the prospect of presumption reversal. For illustrative purposes, we focus on the current Australian position, with 10 as the MACR and 14 the age at which all children are assigned criminal capacity. This reflects the view that, at age 10, the prior probability of criminal capacity would be virtually zero. Clearly, the court will begin the trial with a reasonable doubt about the defendant’s capacity, so it makes sense for the prosecution to bear the burden of proof. As the age of the child defendant increases from 10 to 14, the prior probability of capacity will increase. According to the theoretical construct appearing in Figure 1, if the increase is linear, the prior probability would hit 25% at 11, 50% at 12 and 75% at 13, before hitting 100% at 14. Over most of the 10- to 14-year interval, the prior probability remains below the high criminal standard of proof. However, at a critical age prior to the child’s 14th birthday, the prior probability will equal and then exceed the standard of proof. The criminal standard does not demand absolute certainty.^89^ Evidence that a child is above this critical age would, prior to the presentation of evidence to the contrary, constitute proof of capacity beyond reasonable doubt. If the criminal standard of proof is at 90%,^90^ for example, with a linear increase, this would occur when the child is roughly 13 years, 7 months and 1 week old. At that point, logically, capacity should be presumed. The implication of the model is that the defence should then be required to establish a reasonable doubt about capacity.

**Figure 2.**
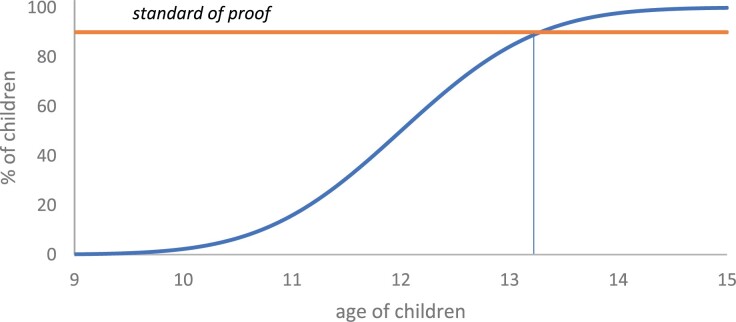
Theoretical construct of percentage of children with capacity: normal distribution.

Objections may be raised to this probabilistic implication. Of course, the age of the presumption reversal in Figure 1 has false precision. It is based on contestable assumptions regarding the probabilistic level of the standard of proof and the rate at which children gain capacity with age. Even if the criminal standard can be understood probabilistically,^91^ questions arise as to its precise level^92^ (putting aside its possible flexibility). And the upper and lower ages are empirically contestable, as is the curve between them. If, for example, we stick with 10 and 14 but replace a linear increase with a normal increase, the prior probability would cross the 90% level about four months earlier. (A theoretical construct appears in Figure 2.)^93^ In addition to these theoretical and empirical issues, the late presumption reversal may appear overly complex. The law only recognises two standards: balance of probabilities and *beyond* reasonable doubt.^94^ Currently, there is no recognised burden of establishing a reasonable doubt.

However, these objections are not fatal. Criminal procedure necessarily operates in the absence of a perfect understanding of matters like child development. And the complexity of a reverse-burden defence may be a price worth paying to better achieve justice.^95^ As discussed in section 2, there is strong scientific evidence that children may lack capacity over a longer period than is currently recognised by the law. In Australia, where the rebuttable presumption currently operates from 10 to 14, an incapacity defence (for simplicity, to be proved on the balance of probabilities) could be introduced to operate from 14 to 18. This may be more feasible politically than extending the term of the rebuttable presumption.

## 4. Weighing the Evidence

Section 3 examined the two elements of the presumption of *doli incapax*—the burden of proof and the standard of proof—and argued that the presumption provides a sensible and workable framework for individualised determinations of capacity. The presumption’s graduated approach to proof of capacity is preferable to that in England & Wales and Northern Ireland, which switches, for all children, from no capacity to full capacity on the child’s 10th birthday. The scientific evidence reviewed in section 2 clearly shows children continue to develop their decision-making capacities, at different rates, through their teen years. If the reintroduction of the presumption is politically unviable, a stand-alone incapacity defence should be introduced for children aged from 10 to at least 14 (but preferably 18). If extending the presumption is unfeasible in Australia, it should be supplemented with a reverse-burden incapacity defence for children aged from 14 to 18.

This section examines various types of evidence regarding a child’s capacity that may be available to the parties. This review will promote a coherent approach to the use of evidence, addressing concerns that the presumption is too difficult to rebut or that it may generate inconsistent practices.^96^ This discussion addresses the rebuttable presumption, for example, as it currently operates in Australia for children aged from 10 to 14. However, the evidence and inferences discussed also have application to an incapacity defence.

Before going into detail, it is worth considering the nature of the fact in issue—a child’s capacity. The presumption of *doli incapax* requires the prosecution to prove ‘that the child understood the moral wrongness of the conduct’.^97^ Understanding is a mental faculty that lacks an accessible physical existence and cannot be proven directly. The prosecution may invoke the ‘general maxim … that all evidence is to be weighed according to the proof which it was in the power of one side to produce, and in the power of the other to have contradicted’.^98^ The defendant’s mind is, by definition, in the defendant’s domain. However, this does not necessarily give the defence a proof advantage.^99^ The defendant may or may not have insight into their mental state. The credibility of any self-serving claims by the defendant is liable to be discounted, particularly if the claims occur some time later. And the prosecution may not be at a disadvantage in adducing evidence of the defendant’s mental state.^100^ Direct evidence of capacity is non-existent, but various other types of evidence may be available, including the defendant’s age; evidence regarding the nature of the charged misconduct and any surrounding circumstances; expert evidence relating to the child’s development; admissions; and evidence of the child’s upbringing, education and prior behaviour. These are considered in the following subsections.

### A. The Defendant’s Age

It is not uncommon in Australia for the prosecution to point to the fact that the child defendant is almost 14 and argue that at that age they must have recognised that their behaviour was seriously wrong. This argument proceeds by reference to what is generally the case with children of that age. The older the child, all else being equal, the more likely it is that they appreciated the wrongness of the charged conduct.

This argument may raise the objection that it rests on generalities, with no specific application to the defendant.^101^ Attention should be paid ‘to the intellectual and moral development of the particular child. Some ten-year-old children will possess the capacity to understand the serious wrongness of their acts while other children aged very nearly fourteen years old will not.’^102^ Expressed this way, the objection is not entirely persuasive. Certainly, it is preferable to base inferences on specific evidence rather than on generalities. The more evidence the better,^103^ and more specific evidence may displace the generalised inference. But in the absence of specific evidence, generalised arguments are a necessity.

Moreover, specificity and generality lie on a spectrum. To a degree, all inferences rely on generalisations;^104^ it is just that some generalisations are narrower than others. For example, an inference of incapacity based on evidence that the defendant, although almost 14 years old at the time, suffered from foetal alcohol syndrome and was the victim of physical abuse from his father is based upon a generalisation about the understanding of almost-14-year-old boys with foetal alcohol syndrome who were the victims of physical abuse from their fathers.

While the fact that a child is almost 14 years old may assist the prosecution in rebutting the presumption of *doli incapax*, it cannot do the job by itself. On the contrary, the fact that the child was under 14 at the time is the basis for the presumption. As discussed above, the logic behind this is a little messy. It would be an oversimplification to say the presumption reflects the view that ‘normal’ children between 10 and 14 lack capacity.^105^ The presumption implies that the relative frequency of children with capacity increases with age, from virtually 0% at age 10 to virtually 100% at 14. For the distributions in Figures 1 and 2,^106^ if ‘normal’ is what is true of a majority, then it might be argued that normal children under 12 lack capacity while normal children over 12 possess capacity. A child over the age of 12 (knowing nothing else about the child) would probably have capacity. Reasonable doubt about capacity would persist for children beyond age 12; however, as discussed above, there is an age approaching 14 which (in the absence of other evidence) would logically prove capacity beyond reasonable doubt. Logically, the presumption should reverse at this point.

Currently there is no presumption reversal. The burden remains on the prosecution right up to age 14. Prosecution reliance on the defendant’s age weakens the force of the presumption but is legally, if not logically, insufficient to rebut it.^107^

### B. The Apparent Wrongness of the Defendant’s Misconduct

Another common prosecution argument is that, given the nature of the misconduct, the defendant must have known that what they were doing was seriously wrong. This may raise an objection like that considered in the previous section. The presumption assumes that the defendant committed the charged acts just as it assumes the child’s age. It follows, on this view, that commission, like age, cannot alone be considered to rebut the presumption. Capacity ‘must be proved by the evidence, and cannot be presumed from the mere commission of the act’.^108^ But this objection is unpersuasive. The evidence will generally go beyond mere commission and show that the defendant has engaged in particular misconduct. As a matter of ‘principle’,^109^ and of ‘logic or experience’,^110^ evidence of commission, depending on its details, may support an inference of capacity.

The inference of capacity from commission resembles the familiar inference of *mens rea* from the performance of the *actus reus*. The defendant’s *mens rea* is often inferred directly from the *actus reus*.

If the immediate consequence of an act is obvious and inevitable, the intentional doing of the act imports the intention to produce the consequence. Thus, to suppose that a sane man who wilfully cuts another man’s throat does not intend to do him harm would be absurd.^111^

Care must be taken to ensure that the inference is warranted. It is not enough that a reasonable person would have appreciated the consequence—that would turn serious offences like murder into offences of negligence.^112^ But with appropriate caution, the inference may be open.

The defence may seek to distinguish the inference of capacity from the inference of *mens rea*. The inference of *mens rea* is premised on the notion that the defendant, an adult, *has a basic level of understanding* and so would appreciate the natural and probable consequences of their actions. Unless the adult defendant is exceptional, they would be assumed to have foreseen and intended the harm resulting from their actions. According to this defence argument, the situation with children is totally different. Children generally *do not have a mature appreciation* of right and wrong. Neuroscience research, discussed in Part 2, confirms that children may well lack the capacity to understand the wrongfulness of behaviour and control their actions accordingly. The issue is whether, despite the presumption, the child exceptionally may recognise the wrongness of their actions. Indeed, the defence may argue that the child’s commission of the horrendous act, rather than proving capacity, tends to confirm the child’s lack of understanding.^113^

**Figure 3.**
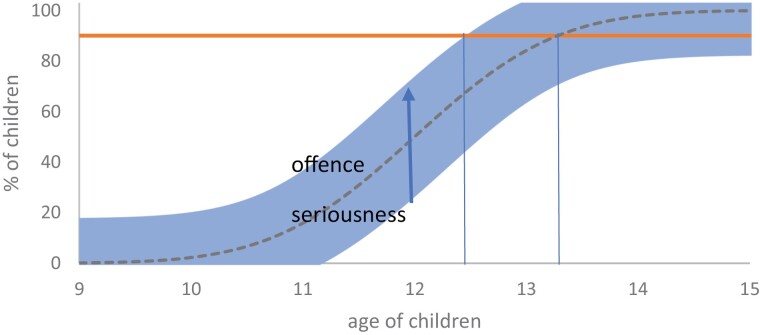
Theoretical construct of percentage of children with capacity: offence seriousness.

This defence argument is ingenious, but it proves too much. People, including children, sometimes do do things that they understand are seriously wrong. A child under 14 may be presumed not to understand the serious wrongness of an offence of average seriousness. However, the more seriously wrong the offence, the more likely the child is to appreciate its wrongness. It follows that where the misconduct was particularly serious, knowledge may be inferred at a younger age. (According to the theoretical construct in Figure 3, capacity for an offence of extreme seriousness is proven at age about 12½, more than a year younger than for an average offence.)

This is not to say that the inference of capacity from commission is straightforward. The initial assessment of the wrongness of the conduct itself poses challenges. Whereas age lies on a numerical scale, the relative wrongness of a criminal offence is a complex, multidimensional issue of fact and value.^114^ The wrongness of causing bodily harm may be more apparent than the wrongness of property damage. Scales may be constructed—for example, the degree of force applied to a victim’s person, or the monetary value of the property that is destroyed.^115^ However, different harms may not lie on the same scale even within a category. An open wound is not immediately comparable with a broken bone, and the wrongness of damage to property may sometimes depend upon its sentimental value to the owner more than its material value.

Other categories of offence pose greater challenges. In one respect, the wrongness of dishonesty offences may be less apparent than property damage offences in that there is a transfer of value rather than its destruction. From another perspective, dishonesty offences are worse in that deceptiveness is superimposed on the victim’s loss,^116^ but this may not be so apparent to a child because it depends upon understanding complex social relations.^117^ Sexual offences raise similar considerations, with the emotional harm potentially obscured behind elaborate social structures. Reading these social situations can be challenging for adults;^118^ the risks of miscommunication and misunderstanding by unsophisticated teenagers, their bodies freshly flooded with hormones, are massively amplified. Sexual offences are among the most serious in the criminal calendar,^119^ but it does not necessarily follow that child defendants will appreciate the seriousness.^120^

### C. The Defendant’s and Victim’s Behaviour Surrounding the Misconduct

The prosecution, in seeking to prove capacity, will often have evidence of the defendant’s behaviour at around the time of the charged misconduct. This may offer genuine insights regarding the defendant’s appreciation of the wrongness of their actions. For example, there may be evidence the defendant planned the offence carefully, which suggests relatively strong intellectual development.^121^ To the extent that there is a correspondence between intellectual development and moral development, this will lend support to the prosecution case that the defendant appreciated the seriousness wrongness of what they were doing. However, the defence may challenge the existence of the correspondence.^122^ This may be the subject of expert evidence (considered in the next subsection).

The prosecution may also argue that the defendant’s appreciation of the wrongness of their actions is revealed by evidence of their subsequent behaviour—for example, their efforts to conceal the offence or deflect blame.^123^ This inference from post-offence conduct resembles the inference of ‘consciousness of guilt’ in the criminal trial. As appreciated in that context, this inference requires careful handling.^124^ The inference relies upon the defendant’s post-offence conduct—concealment or deflection—being more consistent with the guilt theory than the innocence theory. Clarity about the competing case theories is crucial. Where identity is in issue in a murder case, the defendant’s provision of a false alibi may appear incriminating. Why would the defendant lie if he had nothing to do with the killing? The defence may claim that the defendant was seeking to divert unjustified suspicion or to protect the true killer,^125^ but often the lie will appear more consistent with guilt than innocence. However, if the issue is whether the killing was intentional or unintentional—murder or manslaughter—the inference may not be open. There is ‘no hard and fast rule’ precluding the inference.^126^ ‘The result will always turn on the nature of the evidence in question and its relevance to the real issue in dispute.’^127^ But the defence may plausibly argue that the defendant lied to conceal the accidental killing and that the evidence is ‘intractably neutral’ between the competing prosecution and defence theories.^128^ In the capacity context, the defence may similarly argue that the defendant’s post-offence conduct is ‘equivocal in the sense of being equally consistent with an understanding by the [defendant] that his conduct was merely naughty or mischievous’.^129^ Whether an inference of capacity is open will depend upon the circumstances of the case.

The victim’s behaviour at the time of the defendant’s conduct is also relevant to the defendant’s appreciation of the wrongness of their actions. A defence claim that the defendant did not appreciate that it was wrong to sexually interfere with a victim will be harder to sustain where there is evidence that the victim was resisting, crying or screaming.^130^ Despite evidence of the victim’s distress, if the defendant has low cognitive development, it may be unsafe to assume they ‘understand … that the infliction of hurt and distress … involves serious wrongdoing’.^131^

### D. Expert Evidence

The question posed by the *doli incapax* presumption—the level of a child’s moral development in relation to the charged misconduct—may appear tailor-made for expert evidence. However, while potentially useful, expert evidence will be far from definitive.

The issue of the child defendant’s capacity resembles the insanity defence on which expert evidence is generally required and often decisive.^132^ The notion that an expert is not permitted to testify on the ‘ultimate issue’ now lacks support. If such a prohibition ever existed, it has been overturned at common law^133^ and under the Australian uniform evidence law.^134^ Experts have long expressed opinions as to whether defendants satisfy the legal test for the insanity defence.^135^ Notwithstanding the expert’s ‘mantle of expertise’,^136^ the fact-finder is ‘not bound by the expert’s opinion … the issue is for them to decide’.^137^ Having said that, fact-finders ‘must base their conclusions on the *evidence*’,^138^ and a conviction that is contrary to ‘uncontradicted and unchallenged expert evidence’^139^ may be vulnerable on appeal.

Expert evidence could be viewed as less useful with regard to *doli incapax* than it is to insanity. Mental illness is likely to lie outside the experience, knowledge and understanding of the court; expert medical evidence is necessary to fill this gap. By contrast, childhood is a stage ‘through which we must all of us have passed before attaining adulthood and maturity’,^140^ and most of us have observed others go through childhood as well. A child’s development is not ‘a matter … falling exclusively within the domain of scientific expertise’.^141^ Having said that, as discussed in section 2, many children who face criminal charges have not enjoyed a normal background or normal neurodevelopment. In any case, the so-called ‘common knowledge’ rule, if it ever existed, has been abolished. Under the Australian uniform evidence law, experts may provide evidence on ‘matters of ordinary human experience’.^142^ At common law, admissibility depends upon the ‘helpfulness’ of the expert evidence;^143^ ‘whether the evidence will add to the trier of fact’s stock of knowledge so that it can reach a more accurate decision’.^144^ Evidence of a child psychologist may be given ‘significant weight’ on the issue of a child’s capacity,^145^ and courts also accept evidence on the issue from less rarefied experts, such as school teachers.^146^

There is a further respect in which expert evidence may be less helpful regarding *doli incapax* than with mental illness. Mental illness may be more stable and predictable than a child’s understanding of wrongness, which develops over time in conjunction with the child’s neurological development and in response to the child’s experiences.^147^ A mentally ill defendant undergoing treatment may have received a diagnosis prior to the event giving rise to charges. A later diagnosis may provide useful insights into the state of mind of the defendant at the time of the charged conduct.^148^ However, prior psychological assessments may be far less common for child defendants. While many children who face criminal charges will have experienced developmental delays, non-typical neurodevelopment and complex issues, social disadvantage may have hampered opportunities for them to receive professional assessment and intervention.^149^ Assessments conducted after the child’s misconduct may be unhelpful because of the impact of the child having seen the victim’s pain and distress, and been subject to investigation and psychological assessment.^150^

### E. Admissions

The previous subsection noted that an expert opinion based upon observations of the defendant’s behaviour after the charged misconduct may be of limited use because of the impact of the misconduct and its aftermath on the defendant’s understanding. Admission evidence may present a similar problem. Asked whether they knew that their conduct was wrong, it would be natural for the child to answer ‘yes’. The child may well have learnt the wrongness of their actions, or at least have learnt that that is how others view their actions. But that does not necessarily mean they knew their conduct was wrong at the time.

False confessions are a known cause of wrongful convictions.^151^ Children may be particularly susceptible to pressure in questioning and therefore at heightened risk.^152^ The right to remain silent is a fundamental protection for all suspects; it is particularly important to child suspects. Care should be taken to ensure that a child suspect is properly cautioned and understands the caution. In police interviews, tag or leading questions should be avoided.^153^ In *R v McCormick*,^154^ the police officer’s question beginning ‘Did you know that … it was seriously wrong to …’ was considered unhelpful because the wrongness of the child’s behaviour had already been put to him.^155^ An admission made in response to such questions may be subject to exclusion on various grounds, such as having been improperly obtained^156^ or being potentially unreliable and susceptible to misuse.^157^ Even in a properly conducted interview with an appropriate adult present to ‘support, advise and assist them when … they are … asked to provide information’,^158^ a child’s acknowledgement that they understood the wrongness of their actions may be of limited value.

Where the child elects not to answer police questions, care should be taken before inferring this choice displays consciousness of guilt. At common law, the right to silence prohibits this adverse inference; however, this protection has been subject to legislative inroads.^159^ In England & Wales and Northern Ireland, where the adverse inference may otherwise be available, it may be inappropriate. The child’s silence, rather than an effort to avoid true admissions, may be a reasonable strategy against false admissions.^160^

Admissions made by child defendants to people other than police may raise similar issues. The right to silence may not apply in such circumstances.^161^ However, if the child is talking to parents, teachers or other adults, the context may still have the qualities of an interrogation, but without the safeguards. The risk of false admission will remain and there may be grounds for exclusion.^162^

In some respects, admissions made by child defendants to their peers will appear more reliable. The situation may be less pressured and the defendant able to express themselves more freely.^163^ However, the risk that the child’s recognition of the wrongness occurred after the misconduct should still be considered. Further, it would be necessary to consider whether the child was speaking honestly. Depending upon the circumstances, the child may have been boasting and exaggerating.^164^

The defendant also has the right to silence at trial. If the defendant is still a child at trial and elects to testify, as in pre-trial questioning, age-appropriate language should be used in examination and cross-examination,^165^ with the assistance of witness intermediaries,^166^ and any potential admissions should be interpreted with care. If the child elects not to testify, at common law and under Australia’s uniform evidence law, there appears very little scope for any adverse inference.^167^ While the adverse inference may be open in England & Wales and Northern Ireland,^168^ there may be sound reasons not to draw it.^169^ As with silence in the police station, rather than an effort to avoid true admissions, a child’s silence may be a reasonable defence strategy to avoid false admissions.

### F. Upbringing and Antecedents

As discussed, evidence of a child’s admissions and post-offence psychological assessments may be difficult to interpret, in part because the impact of misconduct and its aftermath may obscure the historical enquiry. In this respect, clearer guidance may be obtained from evidence of the child’s experiences preceding the charged misconduct. Courts have recognised that children’s ability to understand the wrongfulness of their behaviour will be influenced by their upbringing.^170^

One potential line of argument would support the defence. A child brought up in a family and community where various forms of criminality are the norm may be less likely to appreciate the wrongness of such behaviour than a child who was brought up in a law-abiding family and community.^171^ In particular, a child’s understanding of the wrongfulness of a sexual offence may be adversely affected by having previously witnessed or been the victim of sexual offences. In *RP v The Queen*, the defendant’s use of a condom to commit sexual assault on his brother was seen as ‘strongly suggestive of his exposure to inappropriate sexually explicit material or of having been himself the subject of sexual interference’.^172^

Evidence that the defendant engaged in this kind of behaviour previously without being reprimanded or punished will support the defence submission that the defendant did not appreciate the wrongness of their behaviour.^173^ However, where the evidence discloses that the defendant was disciplined for the prior misconduct, then the prosecution may be able to invoke the converse argument. Given this history, the defendant would have known their behaviour was wrong.^174^

The defence may object to the admissibility of prosecution evidence of a defendant’s prior misconduct.^175^ Depending upon the jurisdiction, the evidence may be subject to a special exclusionary rule. In England & Wales, the evidence would constitute ‘bad character’ evidence,^176^ but would likely be admissible as ‘important explanatory evidence’.^177^ The evidence would not be caught by the corresponding exclusion in Australia’s uniform evidence law provisions, as the prosecution is not relying on ‘tendency’ reasoning.^178^ The defence may invoke the trial judge’s general power of exclusion;^179^ however, if the inference invited by the prosecution is plausible and probative, it would likely gain admission, particularly in judge-only trials, where the risk of prejudice may appear remote.^180^

A child’s background may have a more general relevance. It should not be assumed that ‘children coming from what used to be called good homes’^181^ necessarily have a better-calibrated moral compass; criminality occurs in all segments of society.^182^ Nevertheless, children that are materially deprived generally face greater developmental challenges.^183^ Such children may suffer a double injustice: the initial social and economic deprivation, and then the criminal law’s failure to acknowledge the impact this may have on their capacity. The law of England & Wales and Northern Ireland is particularly harsh. In assigning full criminal capacity to all children over 10, it discriminates against one of society’s most vulnerable groups.^184^

## 5. Conclusion

Traditionally, for conviction, the law has required child defendants to have criminal capacity. The child must have committed the misconduct despite knowing it was seriously wrong. The common law irrebuttably presumes that children below seven years of age lack capacity and children above 14 years possess capacity. Between these ages, the presumption of *doli incapax* operates, requiring the prosecution to prove capacity beyond reasonable doubt. Different jurisdictions have departed from the common law in various ways. In Australia, the MACR has been raised to 10 and the rebuttable presumption continues to operate to age 14. In England & Wales and Northern Ireland, the MACR has been raised to 10 and the rebuttable presumption has been abolished. At 10 years, children are deemed to possess full criminal capacity. Other common law countries, such as Canada, Ireland and Scotland, have raised the MACR to 12 but have also abolished the rebuttable presumption. Current reform activity in Australia may result in further raising of the MACR, but this may also be accompanied by the abolition of the rebuttable presumption.

Abundant developmental and neuroscience evidence shows that children’s brains are still developing in ways that affect decision-making relevant to criminal responsibility right through the teenage years. The UN Committee has recommended that the MACR be lifted to at least 14 but preferably higher. However, the UN Committee has also opposed the rebuttable presumption as giving courts too much discretion, with discriminatory results. We endorse the lifting of the MACR, but we argue that there is a continuing role for the rebuttable presumption. Many different environmental and biological factors impact on development and children attain capacity at different ages. The law should take account of this; for ages where children may or may not have capacity, the court should consider the evidence relating to the specific child defendant. Further, it is appropriate for the prosecution to prove capacity beyond reasonable doubt. Convicting a child who lacks capacity is a searing injustice; their prospects for a happy and productive life will have been crippled. This is a far worse error than wrongly acquitting a child that had capacity. Requiring the prosecution to prove capacity beyond reasonable doubt minimises the harm flowing from erroneous verdicts.

If reintroduction of the presumption of *doli incapax* is not viable in England & Wales, an alternative is a reverse-burden incapacity defence for children aged from 10 to at least 14 (but preferably 18). The defence should be given the opportunity to prove incapacity on the balance of probabilities. This would not minimise the harm of error as effectively as the rebuttable presumption, but it would still be a vast improvement on current English & Welsh law. In Australia, there is scope for the presumption, which operates between age 10 and 14, to be supplemented by a reverse-burden defence between ages 14 and 18.

Objections that the presumption of *doli incapax* makes a child’s capacity too difficult for the prosecution to prove are overstated. For older children, the presumption weakens, becoming easier to rebut. In particular cases, the prosecution may face evidential and proof difficulties; however, these may arise with regard to many issues. Capacity is hardly unique in that respect. With regard to capacity, a variety of evidence may be available to the prosecution. The prosecution may argue that, given the nature of the misconduct, the defendant must have known it was wrong. The defendant’s behaviour surrounding the misconduct—evidence of planning or concealment—may lend further support, as may evidence of the victim’s distress. Expert evidence of the child’s development may also be available, together with admission evidence. Evidence that the child has previously been punished for similar misconduct will support an inference that the defendant would have known the wrongness of the charged misconduct.

If evidence of capacity is unavailable, inadmissible or insufficiently strong, the child should be acquitted. The criminal standard of proof is demanding and the acquittal may well be incorrect. However, having regard to the lasting harm that a conviction would do to the child, acquittal is the safest and most just verdict.

## Footnotes

1
*RP v R* (2016) 259 CLR 641, 650 (Kiefel, Bell, Keane and Gordon JJ).

2 eg *C v DPP* [1996] AC 1, 38; *RP* (n 1) 649. In *BDO v The Queen* [2023] HCA 16 [24] the High Court noted that, under s 29(2) *Criminal Code* (Qld) the prosecution needs to prove, not actual knowledge ‘but rather the capacity to know or understand’, adding that ‘much of what was said in *RP* … about matters of proof [remains] relevant’: at [23].

3 The term ‘child’ is generally used to refer to a person under 18, in line with art 1 of the UN Convention on the Rights of the Child. In most Australian jurisdictions, a child will be tried in the Children’s Court (except for serious children’s indictable offences) for offences committed while they were aged under 18: eg Children (Criminal Proceedings) Act 1987 (NSW), ss 3, 28(1). Similarly, in England & Wales, a child (person under the age of 14) or a young person (aged 14+ but under 18) will generally be tried in the Youth Court: Children and Young Persons Act 1933, ss 46, 107(1), as amended by the Criminal Justice Act 1991, s 68, sch 8, para 1(3). See also n 22 below.

4 eg E Farmer, ‘The Age of Criminal Responsibility: Developmental Science and Human Rights Perspectives’ (2011) 6 J Child Serv 86, 88–9.

5 This approach is not unique to common law jurisdictions: Thomas Crofts, *The Criminal Responsibility of Children and Young Persons: A Comparison of English and German Law* (Ashgate 2002). For a historical account of the common law position, see AWG Kean, ‘The History of Criminal Liability of Children’ (1937) 53 LQR 364; Thomas Crofts, ‘The Common Law Influence over the Age of Criminal Responsibility—Australia’ (2016) 67 NILQ 283.

6 eg *RP* (n 1) 649, 659.

7 The alternative formulation requiring ‘that the jury must be sure of guilt is now more usual [in England & Wales] … Both are correct’: *Phipson on Evidence* (20th (online) edn, 1 March 2022) [6-51].

8 eg Children and Young Persons Act 1963, s 16(1); Criminal Code (Tas), s 18(1) (as amended by Youth Justice (Consequential Amendments) Act 1999 (Tas), s 3, sch 1).

9 Crime and Disorder Act 1998, s 34; Criminal Justice (Northern Ireland) Order 1998, s 3; *JTB* [2009] AC 1310.

10 eg Criminal Code (RSC, 1985, c C-46), s 13; Criminal Procedure (Scotland) Act 1995, s 41; Children Act 2001 (Irl), s 52(1) as substituted by Criminal Justice Act 2006 (Irl), s 129. In Ireland, a child aged 10 or over but under 12 can be charged with murder, manslaughter or rape, s 52(2). For prosecutions under 14, the permission of the DPP is required, s 52(4).

11 Age of Criminal Responsibility HL Bill (2021–22), 31. A consultation on raising the minimum age of criminal responsibility is currently under way in Northern Ireland, see Department of Justice, Strategic Framework for Youth Justice, 01 March 2022, 47.

12 In February 2019, the Australian Attorneys-General formed a working party to review the age of criminal responsibility. After public consultation, the working party ‘identified the need for further work to occur regarding the need for adequate processes and services for children who exhibit offending behaviour’ and deferred any action: Council of Attorneys-General, *Communique,* 9 December 2022. However, pressure for change in individual States and Territories continues: eg Morag McArthur, Aino Suomi and Belinda Kendall, *Review of the Service System and Implementation Requirements for Raising the Minimum Age of Criminal Responsibility in the Australian Capital Territory: Final Report* (ACT Justice and Community Safety Directorate 2021); Talina Drabsch, *Age of Criminal Responsibility* (NSW Parliamentary Research Service, e-brief Issue 1/2022, March 2022); Queensland Community Support and Services Committee, *Criminal Law (Raising the Age of Responsibility) Amendment Bill 2021, Report No 16, 57th Parliament* (2022). Bills have recently been introduced in a number of Australian Parliaments: Crimes Legislation Amendment (Age of Criminal Responsibility) Bill 2019 (Cth); Children (Criminal Proceedings) Amendment (Age of Criminal Responsibility) Bill 2021 (NSW); Criminal Law (Raising the Age of Responsibility) Amendment Bill 2021 (Qld)); Young Offenders (Age of Criminal Responsibility) Amendment Bill 2022 (SA). The Northern Territory passed the Criminal Code Amendment (Age of Criminal Responsibility) Act 2022 (NT), raising the age of criminal responsibility to 12 and leaving the presumption in place between ages 12 and 14 (s 4, amending ss 38 and 38A of the Code), but it has not yet commenced. At the end of 2022, the Attorney-General of the Australian Capital Territory stated that in 2023 ‘the ACT Government will move a single Bill to raise the minimum age of criminal responsibility to 12, and then to 14 years within the 2 years after that’: ACT Government, Media Release, 3 November 2022.

13
*C v DPP* (n 2) 39.

14 UN Committee on the Rights of the Child, ‘General Comment No 24’ (2019) UN Doc CRC/C/GC/24 (UN Committee (2019)), [27]; UN Committee on the Rights of the Child, ‘Concluding Observations: Australia’ (2005) UN Doc CRC/C/15/Add.268, para 74a; UN Committee on the Rights of the Child, ‘Concluding Observations: Australia’ (2012) UN Doc CRC/C/AUS/CO/4, para 84(a); UN Committee on the Rights of the Child, ‘Concluding Observations on the Fifth Periodic Report of the United Kingdom of Great Britain and Northern Ireland’ (2016) UN Doc CRC/C/GBR/CO/5, 78(a).

15 UN Committee on the Rights of the Child, ‘General Comment No 10’ (2007) UN Doc CRC/C/GC/10, [30].

16 UN Committee (2019) (n 14) [22].

17 ibid.

18 Royal Commission into the Protection and Detention of Children in the Northern Territory (NT Royal Commission), *Final Report* (2017) vol 1, 134.

19 E Cauffman and L Steinberg, ‘(Im)maturity of Judgment in Adolescence: Why Adolescents May Be Less Culpable than Adults’ (2000) 18 Behavioral Sciences & the Law 741, 759.

20 Sentencing Advisory Council of Victoria, *Sentencing Children and Young People in Victoria* (2012).

21 JE Nurmi, ‘How Do Adolescents See Their Future? A Review of the Development of Future Orientation and Planning’ (1991) 11 Developmental Review 1; A Benthin, P Slovic and H Severson, ‘A Psychometric Study of Adolescent Risk Perception’ (1993) 16 Journal of Adolescence 153; L Furby and R Beyth-Marom, ‘Risk Taking in Adolescence: A Decision-Making Perspective’ (1992) 12 Developmental Review 1; L Steinberg and K Monahan, ‘Age Differences in Resistance to Peer Influence’ (2007) 43 Developmental Psychol 1531.

22 UN Standard Minimum Rules for the Administration of Juvenile Justice 1985 (the Beijing Rules) commentary to R 4.1.

23 eg Marriage Act 1961 (Cth) ss 11, 12, which permits marriage at 16 with a court order. In England & Wales, it is no longer possible to marry below the age of 18: Marriage Act 1949 (UK), s 2 as amended by Marriage and Civil Partnership (Minimum Age) Act 2022, s 1.

24 UN Committee (2019) (n 14) [26].

25 ibid [27].

26 The Royal Society, *Brain Waves Module 4: Neuroscience & the Law* (2011) 13.

27 Nicholas J Lennings and Chris J Lennings, ‘Assessing Serious Harm under the Doctrine of Doli Incapax: A Case Study’ (2014) 21 Psychiatry, Psychology and Law 791, 793.

28 Royal Society (n 26) 3–4; M Rutter et al, ‘Integrating Nature and Nurture: Implications of Person Environment Correlations and Interactions for Developmental Psychopathology’ (1999) 92 Development & Psychopathology 335. For discussion, see Thomas Crofts, Enys Delmage and Laura Janes, ‘Deterring Children from Crime through Sentencing: Can It Be Justified? (2022) Youth Justice, DOI: 10.1177/14732254221104896.

29 The Centre for Social Justice, *Rules of Engagement: Changing the Heart of Youth Justice* (2012) 201, 202.

30 McArthur, Suomi and Kendall (n 12) 18.

31 NT Royal Commission (n 18) vol 1, 135.

32 ibid.

33 eg NE Dowd, ‘Children’s Equality Rights: Every Child’s Right to Develop to Their Full Capacity’ (2020) 41 Cardozo L Rev 1367.

34
*Phipson on Evidence* (n 7).

35 NT Royal Commission (n 18) vol 1, 9 and vol 2B, 419. Of course, children with capacity may also be harmed by contact with the criminal justice system—as may adults, for that matter. But children without capacity would generally be more vulnerable, and there is no basis for this harm to be imposed.

36
*AL v The Queen* [2017] NSWCCA 34 [149].

37 eg *R (Ali) v Secretary of State for Justice* [2013] 1 WLR 3536 (DC), 3543 [14], a case concerning statutory compensation for a miscarriage of justice: ‘When will a person be demonstrated to be clearly innocent …? … [The] determination [of this question] will depend on the particular facts and circumstances. It is not a matter which is susceptible of a generic answer.’

38
*Re Winship* 397 US 358 (1970), 370.

39
*Van der Meer* (1988) 82 ALR 10, 31 (Deane J); see also *Re Winship* (n 38) 371–2 (Harlan J).

40 This perspective on the criminal standard of proof may be ahistorical: see eg discussion of sources in *Dookheea* (2017) 262 CLR 402, 420–2.

41
*Woolmington v DPP* [1935] AC 1 481–2 (Viscount Sankey); see also *Environmental Protection Authority v Caltex Refining Co Pty Ltd* (1993) 178 CLR 477, 501 (Mason CJ and Toohey J).

42 Above n 2.

43 ibid 10.

44
*Ostrowski v Palmer* (2004) 218 CLR 493, 500 (Gleeson CJ and Kirby J).

45
*Reniger v Fogossa* (1552) 1 Plowden 19, 75 ER 30–1.

46
*C v DPP* (n 2) 33 (emphasis in original).

47 eg G Williams, ‘Criminal Responsibility of Children’ [1954] Crim LR 493, 495.

48
*C v DPP* (n 2) 11.

49 Home Office, *No More Excuses—A New Approach to Tackling Youth Crime in England and Wales* (Cm 3809, 1997) [9].

50 see Crofts, *Criminal Responsibility of Children and Young Persons* (n 5) 65ff.

51 NT Royal Commission (n 18) vol 2B, 419.

52 see J Bernberg and M Krohn, ‘Labeling, Life Chances and Adult Crime: The Direct and Indirect Effects of Official Intervention in Adolescence on Crime in Early Adulthood’ (2003) 41 Criminology 1287; L McAra and S McVie, ‘Youth Justice? The Impact of System Contact on Patterns of Desistance from Offending’ (2007) 4 Eur J Criminol 315; Queensland Family and Child Commission, *The Age of Criminal Responsibility in Queensland* (Queensland Government 2017) 16–23.

53 NT Royal Commission (n 18) vol 2B, 419.

54
*M’Naghten’s Case* (1843) 8 ER 718; *Woolmington* (n 41) 481.

55 Andrew Ashworth and Meredith Blake, ‘The Presumption of Innocence in English Criminal Law’ [1996] Crim LR 306. With common law defences other than insanity, the defence is assigned the evidential burden: Paul Roberts and Adrian Zuckerman, *Criminal Evidence* (2nd edn, OUP 2010) 227–8. This merely requires the defence to point to evidence persuading the trial judge that the defence is a genuine issue. The prosecution is then required to negate the defence beyond reasonable doubt. The evidential burden primarily serves the goal of efficiency, saving the court from spending time on non-issues, and does not reflect a significant reassessment of the relative error costs.

56 eg *Re H* [1996] AC 563, 586; *Schellenberg v Tunnel Holdings* (2000) 200 CLR 121 [123] (Kirby J); *Royal Mail Group Ltd v Efobi* [2021] 1 WLR 3863 [29]. This standard may pose challenges in causation cases: eg *Sienkiewicz v Greif (UK) Ltd* [2011] 2 AC 229 [156] (Lord Rodger); *Amaca Pty Ltd v Ellis* (2010) 240 CLR 111.

57
*Re Winship* (n 38).

58 eg Mental Health (Forensic Provisions) Act 1990 (NSW), s 39; Criminal Procedure (Insanity) Act 1964 (UK), s 5; RD Mackay and G Kearns, ‘More Fact(s) about the Insanity Defence’ [1999] Crim LR 714, 723–4.

59 see eg J Hobson and others, ‘Are Police-Led Social Crime Prevention Initiatives Effective? A Process and Outcome Evaluation of a UK Youth Intervention’ (2018) 20 International Criminal Justice Review 1; McArthur, Suomi and Kendall (n 12) 26-30.

60
*Reniger* (n 45).

61 eg *Chaulk* [1990] 3 SCR 1303, 1342 (Lamer CJ).

62
*Neat Holdings Pty Ltd v Karajan Holdings Pty Ltd* (1992) 67 ALJR 170; *Re B* [2009] 1 AC 11; *Re D* [2008] 1 WLR 1499; see also nn 74–8.

63 eg *R (N) v Mental Health Review Tribunal (Northern Region)* [2006] QB 468, 485–503. In *Morrison v Jenkins* (1949) 80 CLR 626, the ‘whose baby’ case, it was suggested that the applicants would have to exclude ‘all reasonable doubt’ to gain custody of the child: 648.

64
*Bater v Bater* [1951] P 35, 36–7 (Denning LJ); cf *Re D* (n 62) 1514 (Lord Brown). See also Erik Lillquist, ‘Recasting Reasonable Doubt: Decision Theory and the Virtues of Variability’ (2002) 36 UC Davis L Rev 85.

65 Judicial College, *Crown Court Compendium* part I (June 2022), [5-1].

66
*Green* (1971) 126 CLR 28, 32–3; approved in *Dookheea* (n 40) 422.

67 Peter Mirfield, ‘How Many Standards of Proof Are There?’ (2009) 125 LQR 31, 37; Mike Redmayne, ‘Standards of Proof in Civil Litigation’ (1999) 62 MLR 167, 183.

68 Roberts and Zuckerman (n 55) 254. We argue that, given the individual variability in the rate of child development, courts should be permitted to make individualised determinations of capacity. However, it does not follow that standards of proof should be individualised.

69 eg J Braithwaite, ‘Rules and Principles: A Theory of Legal Certainty’ (2002) 27 Australian Journal of Legal Philosophy 47; R Ahdar, ‘Contract Doctrine, Predictability and the Nebulous Exception’ (2014) 73 CLJ 39; International Covenant on Civil and Political Rights, Art 15(1). The value of legal certainty, as an incident of the rule of law, may have less application to procedural law than to substantive law; parties have ‘No … vested interest in the course of procedure’: *Republic of Costa Rica v Erlanger* (1876) 3 Ch D 62, 69 (Mellish LJ); *Maxwell v Murphy* (1957) 96 CLR 261, 267 (Dixon CJ). However, procedural predictability is important to efficiency, including early settlement, in both civil and criminal litigation: Criminal Law Review Division, New South Wales Attorney-General’s Department, *Report of the Trial Efficiency Working Group* (March 2009); Anthony Edwards, ‘The Other Leveson Report — the Review of Efficiency in Criminal Proceedings’ [2015] Crim L Rev 399; cf Redmayne (n 67) 182.

70 eg Civil Liability Act 2002 (NSW), s 5B(2)(a), (b). Though Gleeson CJ in *NSW v Fahy* (2007) 232 CLR 486, 491 describes the term ‘calculus’ as ‘unfortunate …’ and warns against an ‘unduly mathematical approach’: see also 525 (Kirby J).

71 ‘A “perverse” verdict of not guilty … has traditionally been seen as a protection against oppressive laws or prosecutions’: *LK* (2009) 241 CLR 177 [31] (French CJ); see also John Jackson, ‘Unbecoming Jurors and Unreasoned Verdicts: Realising Integrity in the Jury Room’ in Jill Hunter and others (eds) *The Integrity of Criminal Process: From Theory into Practice* (Hart Publishing 2016) 261, 291. It is unclear whether the judicial fact-finders of a Children’s Court or Youth Court may legitimately deliver perverse acquittals: see also n 3.

72
*Cth v Brennan* 399 Mass 358, 364 (1987).

73
*Re H* (n 56) 586. See also *Neat Holdings* (n 62) 171; *Re D* (n 62) 1508–9 (Lord Carswell), 1513 (Lord Brown); *Re B* (n 62) 19–21 (Lord Hoffmann), 35–6 (Baroness Hale).

74 While the prior probability theory currently has more judicial support than the variable standard theory advanced, there is no inconsistency between the two: D Hamer, ‘The Civil Standard of Proof Uncertainty: Probability, Belief and Justice’ (1994) 16 Syd LR 506, 513–14.

75
*Neat Holdings* (n 62) 171.

76
*Re S-B* [2010] 1 AC 678 [12]; see also Redmayne (n 67) 184–5; Mirfield (n 67) 33.

77
*Re H* (n 56) 586 (Lord Nicholls).

78 David Hamer, ‘Presumptions, Standards and Burdens: Managing the Cost of Error’ (2014) 13 Law, Probability and Risk 221, 224. The precise rationale of the presumption of innocence remains contested: eg Federico Picinali, ‘The Presumption of Innocence: A Deflationary Account’ (2020) 84 MLR 78.

79 Andrew Ashworth, ‘Four Threats to the Presumption of Innocence’ (2006) 10 E&P 241, 249; Mike Redmayne, ‘Appeals to Reason’ (2002) 65 MLR 19, 29; C McCormick, *On Evidence* (West Publishing 1954) 647.

80 Hamer, ‘Presumptions, Standards and Burdens’ (n 78) 225.

81 see also *Re B* (n 62) [15] (Lord Hoffmann), [73] (Baroness Hale).

82 See nn 26–31 above.

83 ATH Smith, ‘Doli Incapax under Threat’ (1994) 53 CLJ 426, 427.

84 E Landowski, ‘Truth and Veridiction in Law’ (1989) 2 International Journal for the Semiotics of Law 29, 35–6.

85
*MacCormick v Federal Commissioner of Taxation* (1984) 158 CLR 622, 646.

86 ibid.

87
*RP* (n 1) 651.

88
*B* [1958] 44 Cr App R 1, 3; *Whitty* (1993) 66 A Crim R 462 at 465; quoted in *B* [1997] QCA 486; *McCormick* [2002] QDC 343 [10]; *RH v DPP* [2013] NSWSC 520. This has long been recognised. According to Hale ‘if the law requires such an evidence where the offender is above twelve, and under fourteen, [then it requires] much more if he were under twelve at the time of the fact committed’, Matthew Hale, *The History of the Pleas of the Crown*, vol 1 (1736) 26. Contrary to the High Court’s claim in *RP*, this approach is not ‘apt to suggest that children mature at a uniform rate’: (n 2) 651. As discussed in the text, it reflects the relative frequency of children achieving maturity, taking account of variation in the rate of maturation.

89 ‘[A]bsolute mathematical or metaphysical certainty is not essential, and in the course of judicial investigations would be usually unattainable’: *Briginshaw v Briginshaw* (1938) 60 CLR 336, 360 (Dixon J), quoting T Starkie, *A Practical Treatise of the Law of Evidence* (4th edn, V & R Stevens & GS Norton 1853) 817.

90 A standard close to this may be implied by Blackstone’s observation that ‘it is better that ten guilty persons escape than that one innocent should suffer’: William Blackstone, *Commentaries on the Laws of England* (1765) vol 4, c 27, 352; David Hamer, ‘Probabilistic Standards, Their Complements, and the Errors That May Be Expected to Flow from Them’ (2004) 1 University of New England Law Journal 71, 84.

91 Long a matter of academic debate: eg M Finkelstein and W Fairley, ‘A Bayesian Approach to Identification Evidence’ (1970) 83 Harv LR 489; L Tribe, ‘Trial by Mathematics: Precision and Ritual in the Legal Process’ (1971) 84 Harv LR 1329; M Finkelstein and W Fairley, ‘A Comment on “Trial by Mathematics”’ (1971) 84 Harv LR 1801; G Williams, ‘The Mathematics of Proof’ [1979] Crim LR 297, 340; LJ Cohen, ‘The Logic of Proof’ [1980] Crim LR 91; G Williams, ‘A Short Rejoinder’ [1980] Crim LR 103; LJ Cohen, ‘Letter to the Editor’ [1980] Crim LR 747; J Koehler and D Shaviro, ‘Veridical Verdicts: Increasing Verdict Accuracy through the Use of Overtly Probabilistic Evidence and Methods’ (1990) 75 Cornell L Rev 247.

92 eg A Volokh, ‘*n* Guilty Men’ (1997) 146 U Penn L Rev 373.

93 This is a cumulative normal distribution, mean 12, standard deviation 1.

94 ‘It is indisputable that only two standards are recognised by the common law, proof on the balance of probabilities and proof beyond reasonable doubt’: *Re D* [2009] 1 AC 11 [23].

95 eg DM Driesen, ‘Complexity and Simplicity in Law’ (2015) 45 Environmental Law 181, 189.

96 UN Committee (2019) (n 14); Thomas Crofts, ‘Prosecuting Child Offenders: Factors Relevant to the Rebuttal of the Presumption of *Doli Incapax*’ (2018) 40 Syd LR 339, 341, 364–5.

97
*RP* (n 1) 650. Understanding must be established in relation to the actual act committed.

98
*Blatch v Archer* (1774) 1 Cowp 63, 65; 98 ER 969, 970 (Lord Mansfield); *Apollo Shower Screens Pty Ltd v Building & Construction Industry Long Service Payments Corporation* (1985) 1 NSWLR 561, 564 (Hunt J). This consideration has been referred to in relation to insanity and regulatory offences: eg *Chaulk* [1990] 3 SCR 1303, 1337–8, 1342 (Lamer CJ); *Schwartz* [1988] 2 SCR 443, 486 (McIntyre J).

99 David Hamer, ‘The Presumption of Innocence and Reverse Burdens: A Balancing Act’ (2007) 66 CLJ 142, 161–2.

100 eg Australian Law Reform Commission and Human Rights and Equal Opportunity Commission, *Seen and Heard: Priority for Children in the Legal Process* (1997) Report No 84, [18.19].

101 eg ‘since, by its very nature, the statistical evidence does not deal with the individual case, something more will be required’: *Sienkiewicz v Greif (UK) Ltd* [2011] 2 AC 229, 287 (Lord Rodger); see further David Hamer, ‘Probability, Anti-resilience and the Weight of Expectation’ (2012) 11 Law, Probability and Risk 135.

102
*RP* (n 1) 651.

103 Crofts, ‘Prosecuting Child Offenders’ (n 96) 366.

104 see eg Tribe (n 91) 1330, fn 2; Koehler and Shaviro (n 91) 252.

105
*RP* (n 1) 651, 657; see Crofts, ‘Prosecuting Child Offenders’ (n 96) 354–6.

106 And any other distribution where less than 50% of children below 12 have understanding and more than 50% of children above 12 have understanding.

107
*B* (n 88) (Pincus JA).

108
*Smith* (1845) 1 Cox CC 260 (Erle J), quoted in *C v DPP* (n 2) 38 (Lord Lowry).

109
*ALH* (2003) 6 VR 276, 281 (Callaway JA); see also *BP* [2006] NSWCCA 172 [29]–[30].

110
*ALH* (n 109) 298 (Cummins AJA).

111
*Parker* (1963) 111 CLR 610, 649 (Windeyer J).

112 ibid 632 (Dixon CJ); see also *Schultz* [1982] WAR 171, 174 (Burt CJ).

113 Jane Fortin, *Children’s Rights and the Developing Law* (3rd edn, CUP 2009) 688.

114 In *M* (1977) 16 SASR 589, 591, Bray CJ suggested that the ‘test is whether he knew that it was wrong according to the ordinary principles of reasonable men’. ‘[D]etermining states of mind has an inescapable value-judgment component … which changes the ultimate issue from one of fact to one of value’: DM Risinger, ‘Searching for Truth in the American Law of Evidence and Proof’ (2013) 47 Ga L Rev 801, 832; see also M Hoffman, ‘The Myth of Factual Innocence’ (2007) 82 Chi-Kent L Rev 663, 663.

115
*BC* [2019] NSWCCA 111 [53].

116 see *C v DPP* (n 2) 6 (Laws J).

117 Crofts, ‘Prosecuting Child Offenders’ (n 96) 348.

118 Prompting ongoing law reform: eg New South Wales Law Reform Commission, *Consent in Relation to Sexual Offences* (September 2020) Report No 148; Crimes Legislation Amendment (Sexual Consent Reforms) Act 2021 (NSW).

119 eg *Siganto (No 2)* (1999) 106 A Crim R 30; *King* [2011] NSWCCA 46 [19].

120 eg *RP* (n 1).

121 eg in relation to the insanity defence: *Cth v Kostka* 370 Mass 516, 521 (1976); *US v Shackelford* 494 F 2d 67, 72 (1974).

122 E Barandse and others, ‘Social Behaviour and Social Cognition in High-Functioning Adolescents with Autism Spectrum Disorder (ASD): Two Sides of the Same Coin?’ (2018) 19 Cognitive Processing 545.

123 eg in relation to the insanity defence: *Cth v Keita* 429 Mass 843, 848–9 (1999); *Cth v Kappler* 416 Mass 574, 597–8 (1993).

124 eg *Lucas (Ruth)* [1981]1 QB 720, 724; *Zoneff* (2000) 200 CLR 234, 258 (Kirby J); David Hamer, ‘“Hoist with His Own Petard’? Guilty Lies and Ironic Inferences in Criminal Proof” (2001) 54 CLP 377.

125 eg *Zoneff* (n 124) 259 (Kirby J).

126
*Baden-Clay* (2016) 258 CLR 308 [74].

127 ibid, quoting *White* [1998] 2 SCR 72 [32] (Major J).

128
*Ciantar* (2006) 16 VR 26 [40]; *Richens* [1993] 4 All ER 877, 885 (Taylor LCJ).

129
*BC* (n 115) [54]; see also *A v DPP* [1992] Crim LR 34, 35; *C v DPP* (n 2) 39; *JA* (2007) 1 ACTLR 126, 138.

130
*AL* (n 36) [135]–[139], distinguishing *RP* (n 1). See also *A v DPP* (n 129); *BP* (n 109) [30].

131
*RP* (n 1) 658, with reference to the defendant’s ‘intellectual limitations’.

132 Criminal Procedure (Insanity and Unfitness to Plead) Act 1991, s 1(1); *Brennan* [2015] 1 WLR 2060, 2070; New South Wales Law Reform Commission, *People with Cognitive and Mental Health Impairments in the Criminal Justice System: Criminal Responsibility and Consequences* (2013) Report 138, [2.67].

133 Roberts and Zuckerman (n 55) 493, discussing *Stockwell* (1993) 97 Cr App R 260, 265–6; Andrew Ligertwood and Gary Edmond, *Australian Evidence* (6th edn, LexisNexis 2017) 791, citing *Murphy v The Queen* (1989) 167 CLR 94, 110.

134 eg Evidence Act 1995 (Cth), s 80(a).

135 eg *Holmes* [1953] 2 All ER 324; *Bleta* [1964] SCR 561, 565–6.

136
*Morgan* (2011) 215 A Crim R 33, 61.

137
*Stockwell* (1993) 97 Cr App R 260, 265–6.

138
*Brennan* (n 132) 2070 (emphasis in original).

139 ibid.

140
*Camplin* [1978] 1 All ER 1236, 1241 (Bridge LJ).

141
*Brennan* (n 132) 2070. eg ‘Jurors do not need psychiatrists to tell them how ordinary folk who are not suffering from any mental illness are likely to react to the stresses and strains of life’: *Turner* [1975] QB 834, 841 (Lawton LJ).

142 eg Evidence Act 1995 (NSW), s 80(b); *AB (No 2)* (2018) 97 NSWLR 1031 [21].

143 Roberts and Zuckerman (n 55) 486.

144 Ligertwood and Edmond (n 133) 789.

145
*KG v Firth* (2019) 278 A Crim R 249, 260.

146
*JA* (n 129); *BP* (n 109) [18]; *L v DPP* [1996] 2 Cr App R 501.

147 See nn 26–31 above.

148 Although in *Zhang* [2000] NSWSC 1099, in which the defence sought to have police evidence of admissions excluded due to the defendant’s schizophrenia, an expert testified this condition had developed after the police interviews: [62].

149 See Homel and others, ‘Preventing the Onset of Youth Offending: The Impact of the Pathways to Prevention Project on Child Behaviour and Wellbeing’ (Australian Institute of Criminology 2015) Trends & Issues in Crime and Criminal Justice No 481.

150 In *LMW* [1999] NSWSC 1342, the psychiatrist’s report was rejected because it was based on an interview with LMW that took place 19 months after the incident: [11]–[12].

151 eg RA Leo and SA Drizin, ‘The Problem of False Confessions in the Post-DNA World’ (2004) 82 North Carolina Law Review 891.

152 eg A Redlich and others, ‘The Police Interrogation of Children and Adolescents’ in GD Lassiter (ed), *Interrogations, Confessions, and Entrapment* (Springer 2004) 107; D Brown and M Lamb ‘Can Children Be Useful Witnesses? It Depends How They Are Questioned’ (2015) 9 Child Development Perspectives 250.

153 See eg Inns of Court College of Advocacy, ‘Advocacy for Vulnerable People and Children National Training Programme: The 20 Principles of Questioning A Guide to the Cross-Examination of Vulnerable People and Children’ (2022); JM Wheatcroft and S Woods, ‘Effectiveness of Witness Preparation and Cross-Examination Non-directive and Directive Leading Question Styles on Witness Accuracy and Confidence’ [2010] 14 E&P 189; S Garven, JM Wood and RS Malpass, ‘Allegations of Wrongdoing: The Effects of Reinforcement on Children’s Mundane and Fantastic Claims’ (2000) 85 Journal of Applied Psychology 38; C Hughes-Scholes and M Powell, ’An Examination of the Types of Leading Questions Used by Investigative Interviewers of Children’ (2008) 31 Policing 210.

154 Above n 88.

155 ibid [20]–[21].

156 eg Evidence Act 1995 (NSW), ss 84, 90, 138; Police and Criminal Evidence Act 1984 (PACE), ss 76(2)(a), 78.

157 eg Evidence Act 1995 (NSW), ss 85, 90, 137; PACE, ss 76(2)(b), 78.

158 PACE, Code C, s 1.7A; see also ss 3.13–3.15; Law Enforcement (Powers and Responsibilities) Regulation 2016 (NSW), regs 28(1)(a), 31.

159 The common law position is stated in *Petty* (1991) 173 CLR 95, 99 and *Director of Serious Fraud Office, Ex p Smith* [1993] AC 1, 30–1. This was adopted in the uniform evidence law: eg Evidence Act 1995 (NSW), s 89. The adverse inference is, however, permitted in some circumstances under legislation: Criminal Justice and Public Order Act 1994 (CJPOA), s 34; Evidence Act 1995 (NSW), s 89A. In NSW, the adverse inference is not available against defendants who were under 18 at the time of questioning (s 89A(5)(a)); however, the CJPOA contains no such limitation.

160 CJPOA, s 34(1).

161 The right is limited to questioning by persons ‘in authority’: *Petty* (n 159) 99; *Ex p Smith* (n 159) 30–1. Arguably, this could extend to questioning of a child by teachers or parents.

162 See nn 156–7, but note the restrictions in Evidence Act 1995 (NSW), s 85(1).

163 See eg *RH v DPP* (n 88) [31]–[34].

164 Crofts, ‘Prosecuting Child Offenders’ (n 96) 364.

165 eg Evidence Act 1995 (NSW), s 41; J Plotnikoff and R Woolfson, ‘Cross-Examining Children—Testing not Trickery’ (2010) 7 Archbold Review 7.

166 Intermediaries are generally available to children accused of crime in more limited circumstances than child victims or child witnesses. See Raymond Arthur and Thomas Crofts, ‘The Use of Intermediaries for Young Defendants: Overcoming Barriers to Young People’s Participation in Criminal Proceedings’ (2022) 34(2) Child & Family Law Quarterly.

167
*Bathurst* [1968] 2 QB 99, 107–8 (Lord Parker CJ); *Azzopardi* (2001) 205 CLR 50, 64 (Gaudron, Gummow, Kirby and Hayne JJ); Evidence Act 1995 (NSW), ss 20(2); cf *Weissensteiner* (1993) 178 CLR 227–8 (Mason CJ, Deane and Dawson JJ).

168 CJPOA, s 35. As originally passed, the provision did not apply to children under 14 (s 35(1)), but this restriction was removed in 1998: Crime and Disorder Act 1998, s 35.

169 CJPOA, s 35(2).

170 eg *AL* (n 36); *RP* (n 1); *F v Padwick* [1959] Crim LR 439.

171 R White, *The Abnormal Personality* (3rd edn, Ronald Press Co 1964) 357; see also J Cowell and J Decety, ‘Precursors to Morality in Development as a Complex Interplay between Neural, Socioenvironmental, and Behavioral Facets’ (2015) 112(41) Proceedings of the National Academy of Sciences 12657; J Smetana, M Jambon and C Ball, ‘Normative Changes and Individual Differences in Early Moral Judgments: A Constructivist Developmental Perspective’ (2018) 61 Human Development 264.

172
*RP* (n 1) 657.

173 eg ibid 652–3.

174 eg *BP* (n 109) [18].

175 eg *C v DPP* (n 2) 34 (Lord Lowry).

176 Criminal Justice Act 2003, s 98.

177 ibid ss 101(1)(c), 102.

178 eg Evidence Act 1995 (NSW), ss 97(1), 98(1); see *M* (1977) 16 SASR 589; *GW* (2015) 20 DCLR(NSW) 236, 240–1.

179 eg Evidence Act 1995 (NSW), ss 101, 137; *Christie* [1914] AC 545; PACE, s 78.

180 Probative value would be weighed against prejudicial risk. Where children are tried without a jury, the risk of prejudice would be viewed as far lower.

181
*C v DPP* (n 2) 11 (Laws J); cf G Williams, *Criminal Law, The General Part* (2nd edn, Stevens 1961) 819.

182 The relationship between class and crime is a topic of perennial interest: eg B Miller, ‘Social Class and Crime’ in W Jennings (ed), *The Wiley Encyclopedia of Crime & Punishment* (Wiley-Blackwell 2015).

183 V McCloyd, ‘Socioeconomic Disadvantage and Child Development’ (1998) 53 American Psychologist 185; NT Royal Commission (n 18) vol 1, 134–40.

184 See n 33 above.

